# Clinical Practice of Nursing Students in South Korea’s Community Treatment Centers During COVID-19: A Descriptive Phenomenological Study

**DOI:** 10.3390/healthcare13222829

**Published:** 2025-11-07

**Authors:** Yungyong Jeon, Chung-uk Oh, Misook Park, Seunyoung Joe, Eunji Kwon

**Affiliations:** 1 Department of Nursing, Korea Armed Forces Nursing Academy, 90 Jaun-ro, Daejeon Metropolitan City 34059, Republic of Korea; tjjyg2@mnd.go.kr (Y.J.); anjoe116@mnd.go.kr (S.J.); a15-10221@mnd.go.kr (E.K.); 2 Department of Nursing, Kangwon National University, 346 Hwangjo-gil, Dogye-eup, Samcheok-si 24341, Gangwon-do, Republic of Korea; 3 Department of Nursing, Korea National University of Transportation, 61 Daehak-ro, Jeungpyeong-gun 27909, Chungcheongbuk-do, Republic of Korea; mspark@ut.ac.kr

**Keywords:** COVID-19, community treatment centers, nursing students, descriptive phenomenology, disaster nursing, South Korea

## Abstract

**Background/Objectives**: This study explored the lived experiences of nursing students in South Korea who participated in clinical practice at Community Treatment Centers (CTCs) during the COVID-19 pandemic. **Methods**: This study was designed as a qualitative study and applied Colaizzi’s descriptive phenomenology. Semi-structured interviews were conducted with ten nursing students who practiced at CTCs for three to four weeks. Data were analyzed through Colaizzi’s seven procedural steps to derive the essential structure of their experience. Data saturation was achieved, and methodological rigor criteria were applied. **Results**: Four overarching themes emerged: (1) transformative growth through immersive clinical practice in quarantine; (2) enduring and adapting to uncertainty and emotional turmoil; (3) reconciling vulnerability and responsibility as future professionals; and (4) validation and pride in becoming visible during a national crisis. **Conclusions**: The study revealed that CTC practice constituted a transformative learning experience that enhanced students’ professional identity and resilience in disaster situations. Findings highlight the need to integrate disaster ethics and psychosocial preparedness into undergraduate nursing curricula.

## 1. Introduction

### 1.1. COVID-19 and CTCs in South Korea

The coronavirus disease 2019 (COVID-19) is an emerging respiratory infectious disease and has been spreading worldwide [1]. To address the shortage of dedicated hospitals for confirmed cases, community treatment centers (CTCs) were temporarily operated in South Korea, the first non-hospital settings with medical oversight for mild or asymptomatic COVID-19 patients during isolation, and often established in repurposed facilities such as training centers, dormitories, or public buildings since 2020 [2]. The Korean government authorized the deployment of nursing students to replenish the lack of nursing staff on CTCs. The nursing activities on CTC fields were non-face-to-face body temperature measurement, symptom monitoring, medication, PCR tests, and transition care to the hospital when necessary.

### 1.2. Clinical Practice During Pandemic

The COVID-19 pandemic has had a significant impact on nursing education. Many nursing schools in Korea have replaced clinical practice with on-campus lab training or conducted it remotely, citing student protection and infection prevention [3]. However, these alternatives are known to impact academic achievement and satisfaction, as they fail to deliver the learning content through vivid experiences, leading to anxiety about future competencies as licensed nurses and careers [4]. Some countries have deployed nursing students to COVID-19 sites as aides, clinical support workers, or students in hospitals to address nursing shortages and complete statutory practicum hours [5,6,7]. While this is somewhat consistent with the ICN’s policy of fostering desirable disaster nursing competencies at bachelor’s degree courses [8], deploying unlicensed students to disaster sites presents a challenge. Clinical practice in a hostile environment can lead to negative emotions such as stress and anxiety, and in severe cases, this can lead students to a loss of self-identity and nursing identity, leading to doubts about the future of nursing [9].

### 1.3. Research Gap and Study Rationale

Just as nurses experience significant mental and psychological stress in COVID-19 settings [10,11,12,13,14,15,16], students have also been reported to experience dissonance between the pressure of infection and their sense of calling to help others [5,6,7]. However, these studies focused on hospital settings, where clinical practice typically takes place. In contrast, CTCs are unique in that they are non-hospital, much of the work is non-contact, and quarantined patients often express strong emotional frustrations to medical staff in response to restrictions on their rights [17,18]. Therefore, it is necessary to explore how these contextual factors impact student clinical practice learning and identity formation.

## 2. Materials and Methods

### 2.1. Study Design

This study was designed as a qualitative study and applied descriptive phenomenology (Colaizzi, 1978) [19] to understand the clinical practice experience of nursing students in the CTCs during the COVID-19 pandemic in South Korea. Within this study, we deeply explored from a phenomenological standpoint, the unique experiences of nursing students practicing in CTCs during COVID-19—to understand their emotions, challenges, coping mechanisms, and the formation of professional identity under crisis conditions.

### 2.2. Context and Setting

The Korean government considered replacing some of the insufficient nursing personnel with nursing students based on Medical Service Act of South Korea [20]. Medical Service Act states that healthcare services can be performed by nursing students under the guidance and supervision of medical personnel during national disaster situations. The Korean government held a meeting with the heads of nursing colleges. There was one nursing school that responded to the government’s proposal. Nursing students and their parents have been explained the necessity of deployment to CTCs and provided a Q&A session. The school office monitored to see if any parents or students disagreed, but none did. Student preferences were actively reflected, with requests for preferred practice mates and allocation order being accommodated. To provide immediate teaching required for practice at CTCs and to respond to potential safety incidents, each CTC was assigned with school faculty member with nursing license. In addition, an emergency response headquarters was established at the school to receive reports of unexpected situations occurring at practice sites, procure daily necessities, and periodically monitor students’ health.

The participants were completed the second semester of their third year, including courses “Disaster Emergency Nursing Training”, clinical practices over 500 h. Before starting the practice at CTCs, the professors conducted a two-day preparatory education of personal/equipment protective equipment (PPE/EPP) donning/doffing, ethical decision and role conflict during disaster and invited a nurse working in a CTC to give students vivid experiences. During the clinical practice, the students assisted medical team and managed the patients in person or telemedicine 8 h a day, 5 days a week for 3–4 weeks. The students strictly followed the lifestyle guidelines set by the medical staff. The practice was conducted according to a three-shift work schedule similar to nurses’ schedules. Although the program was conducted as a curriculum, students were provided with the same hazard pay as nurses. No confirmed cases occurred among the students, and a certified psychiatric nurse provided a debriefing session to provide psychiatric support.

### 2.3. Participants

The participants were recruited using the purposive sampling technique. Hard copies were posted on the school’s student bulletin board for 4 weeks, and soft copies were notified online. The contents of the post included the purpose of the study and the timing of the study, the research method (interview type and time required), the name and contact information of the chief researcher, the confidentiality of the participants, and the use of expected research results. Participants applied directly to the professor or contacted them via email or mobile phone handled by chief researcher scheduled an interview with those willing to participate.

The total number of clinical practice experiences in CTCs was 77, of which 10 were applicants. In this study, the subjects were judged to be a highly homogeneous group, having received education at the same educational institution for the past three years and employed at the same medical institution after graduation. Therefore, in accordance with Kuzel (1999)’s recommendation of a sample size of 5–8 for data collection in a homogeneous group [21], the minimum number of participants was set at eight. The researcher predefined that data saturation would be declared if no additional statements were identified in the final two interviews, resulting in a total of 10 participants being recruited.

### 2.4. Data Collection

Data was collected by a semi-structured in-depth interview to minimize the interviewers’ presumption. The questionnaire for this study consisted of sociodemographic questions, including gender, age, the name of CTCs, as well as a key open-ended key questions: “Can you describe your experience in clinical practice at the CTCs and how was it different from the previous clinical practice environment?”. Additional questions were also used to further explore participants’ responses.

Interview protocol tested at two induction meetings and using one pilot interview. Two researchers who were not affiliated with the students’ institution but had expertise in phenomenological study conducted semi-structured individual interviews via videoconferencing (Zoom). All interviews were conducted in Korean and recorded using Zoom. Interviews were undertaken in February 2022 and last between 45 and 75 min. After each interview, the interviewer made field notes and shared them with the researchers for analysis and storage.

### 2.5. Data Analysis

Audio recordings were transcribed using the ClovaNote application. The completed transcripts were cross-checked by three researchers who had not conducted the interviews to ensure accuracy. Data analysis was conducted manually by all five researchers, following Colaizzi’s seven-step phenomenological method [19]. The team first read all transcripts multiple times to grasp a holistic understanding of each narrative. Through reflective reading, 82 meaning units were identified, consolidated into 16 sub-themes, and further refined into four overarching themes. An exhaustive description was then formulated, and the essential structure of the lived experiences was articulated.

An external audit was not performed. However, methodological rigor was maintained through multiple strategies: reflexive memoing, investigator triangulation, and member checking. Prior to data collection, the researchers engaged in reflective journaling to critically examine their own assumptions, particularly with respect to potential faculty–student power dynamics. Several debriefing sessions were held during the study to discuss reflexive insights, challenge bias, and consider alternative interpretations. In addition, two participants reviewed the synthesized findings to confirm the accuracy and completeness of interpretations, ensuring their perspectives were faithfully represented. All data were generated and analyzed in Korean, then translated into English by a professional qualitative research translation agency. The translated data were subsequently reviewed for semantic accuracy by one researcher with a master’s degree from the United States.

### 2.6. Validation and Rigor

To ensure the quality of our qualitative research, we followed Lincoln and Guba (1985) trustworthiness criteria [22]: (1) credibility: the interview transcripts and analysis results were shared with two participants to confirm alignment with their experiences. Researchers independently coded the transcripts, and discrepancies were resolved through discussion and consensus for investigator triangulation. A total of four versions of the codebook were created to document coding decisions, which served as the audit trail throughout the analysis process; (2) transferability: we recruited participants who could provide vivid experiences and rich narratives, and we continued data collection until saturation was achieved to support thick description; (3) dependability: detailed records were maintained, including interview transcripts, coding processes, and analytic decisions, to ensure transparency. The same interview guide was applied consistently across all participants; (4) confirmability: To minimize the influence of researcher bias on the results, we continuously reflected on the thoughts and assumptions that arose during the research process. This ensured that the conclusions reached reflected the voices of the participants.

### 2.7. Ethical Consideration

Informed consent was obtained directly by the chief researcher, who was not affiliated with the participants’ institution, to prevent any potential coercion. Before the study began, the researcher explained the purpose of the research and the interview process. Only those who fully understood the information and voluntarily agreed to participate were asked to sign the consent form. Participants were informed that they could withdraw from the study at any time without penalty.

## 3. Results

We identified 82 meaning units that consolidated into 16 subthemes and 4 overarching themes (Table 1).

### 3.1. Participants Characteristics

The characteristics of participants are as follows (Table 2). The participants had clinical practice for 3–4 weeks at five CTCS. Nine participants were women, and one was man. Four were assigned to CTC A, three to B, one to C, two to D, and none to E. The weeks of practice completed differ depending on the individual schedule of the students and the order of deployment to the CTCs.

### 3.2. Emerging Themes

4 themes and 16 sub-themes related to the participants’ clinical practice experiences at the CTCs were identified (Table 2).

Theme 1: Transformative growth through immersive clinical practice in quarantine

Nursing students described the CTC experience as an immersive and transformative process that allowed them to transcend fear, discover confidence, and envision themselves as future professionals.

Subtheme 1.1. Navigating nursing in a non-contact environment

The participants met the patients through a glass wall at the CTCs, where non-face-to-face treatment was perform252~ed. Vital sign measurement, practiced by nursing students, was not carried out directly, but the results recorded by the patients were indirectly checked from the phone or an App: “The medical team I belonged to communicated through transparent glass windows without any contact with patients.” (P#1); “Since I couldn’t contact the patients for their vital signs, I called them by phone. So, I got to know their blood pressure and body temperature, etc., and there was a thing like registering on an app” (P#2).

Subtheme 1.2. Stepping into the heart of infectious disease response

The participants stated that they felt as if they were dispatched to a disaster site even though it was a clinical practice and expressed that they were able to experience the real situation of disaster response while checking the national quarantine measures and the health authorities’ response to the emerging infectious disease at the site: “I think I have learned a lot more meaningfully because I have studied a lot about the systematic aspects of the quarantine measures and how the health authorities were responding in such a disaster situation” (P#1); “Even for a nursing student, this was an experience that was not easy to obtain, and going directly to the disaster site was a great experience in itself.”(P#6).

Subtheme 1.3. Bearing the emotional weight of quarantine care

The participants felt various emotions in their interactions with the patients, such as anger, gratitude, resent, or absurdity. Some patients expressed their gratitude to the students for coming to the CTC, and dissatisfaction with the restrictions on autonomy due to the isolation. The participants were baffled or felt hurt not to resolve the patient’s demands: “I’m helping out right now, but there are people who are getting annoyed with nurses” (P#1); “Some people asked to provide them a chocolate pie right now because they cannot get out the room. It was a little difficult and baffling to deal with people who make such requests that we could not fulfil” (P#3).

Subtheme 1.4. Finding oneself and growing to another level

The participants stated that the clinical practice at the CTC was rewarding and valuable. In particular, the participants said that although it was a bit risky to care for patients with confirmed COVID-19, the special experience gave them confidence that they could handle anything in the future with a positive attitude: “I think I have received a good clinical education, and based on that, I gained confidence that I would be able to do well anywhere in practice” (P#4); “I’m confident that I’ll be able to do well in my future practice and anywhere else based on this experience”(P#7).

Subtheme 1.5. Discovering professional identity through crisis

In contrast to the existing clinical practice that ends only with observation, at the CTC, the participants sometimes participated in the same tasks as the nurses did, expressing that the participants were more proactive and responsible for this practice. In addition, for the first time, they worked three shifts, including night, and could imagine their future life as a nurse. Therefore, they performed similar tasks with the same work schedule as a nurse, expressing that they were students but felt like they had become nurses: “Although my workload was much less than that of a nurse, I still felt like I was doing the same job, even during the national crisis” (P#5); “As a student doing the same work the nurses did, I thought that I become a real nurse. Like a little practice? It was a new experience” (P#8).

Theme 2: Enduring and adapting to uncertainty and emotional turmoil

Students described struggling with fear of infection, social isolation, and emotional exhaustion, but gradually learned to adapt through peer support, humor, and small moments of relief.

Subtheme 2.1. Overwhelmed by the threat of infection

The participants experienced anxiety and fear of becoming infected while practicing at a CTC with a new infectious disease for which the treatment is not yet known. To avoid infection, they reported that they avoided direct contact with the patients as much as possible or would make sure to wear protective. One participant reported that she/he was too nervous and fainted while caring for a patient in protective clothing: “I had a strong will to not get infected with COVID-19. There were many heaters in the room, and I stood in front of the heater for a while. So, on the first day, 30–40 min after I put on PPE/EPP, I fainted” (P#5); “I was a little scared, so I put on my goggles and such too tightly, and as soon as I went in, my head started hurting so much that I thought I was going to collapse” (P#7).

Subtheme 2.2. Living under double restrictions as students and medical workers

The participants experienced double restrictions because of the guidelines to be followed as a CTC worker and their role as a nursing student. As a staff, even taking a walk was not allowed due to concerns about overlapping contact tracing with confirmed patients in some CTCs. The participants expressed their frustration and a relative sense of deprivation of having to isolate themselves from society with sick patients, even though they were students: “I was so frustrated because I was always stuck inside and going back and forth between my dorm room and work every day” (P#1); “We were still working here, and the situation outside didn’t seem to be getting any better, and I was seeing my friends from other schools playing and doing so well through social media” (P#5).

Subtheme 2.3. Anxiety in the face of uncertainty

As for the sudden decision to be placed in the CTCs, no one gave the participants specific information about the duration and type of work they would do in the center, resulting in them experiencing anxiety amid uncertainty: “No one told me where the end was. How long do I have to do this, and how long do I have to live this life? It was so confusing in the middle” (P#2); “At first I thought it was a little unbelievable and a little scary” (P#6).

Subtheme 2.4. Finding moments of relief and connection

The participants overcame the stress of life restrictions and uncertain dispatch situations in various ways. Some participants found comfort by talking with their roommates and doing small daily activities such as birthday parties. In addition, they received strength from the support of family, friends, professors, CTC’s staff, and their seniors/juniors as well as material support from various institutions: “Decorating the bulletin board to welcome the new year and eating cup ramen with friends while looking at the snow was a great comfort” (P#3); “I was quite tired because I was mentally and physically less active than usual. But at that time, it snowed, so I went downstairs and made a small snowman, watched the snow, stepped on it, so it was fun” (P#9).

Subtheme 2.5. Finding myself getting used to it

At the CTC, which started with anxiety and vague emotions at first, the participants expressed that their work became easy and that they found themselves gradually adapting to it. Some participants expressed that they were afraid at first, but when they came to the field, they wanted to be helpful and to do well: “As I settled down, I adapted to the life there” (P#2); “We also had some fearful feelings, but after being put on the scene, we had a little fiery emotion” (P#4).

Theme 3: Reconciling vulnerability and responsibility as future professional

Students experienced internal conflict between their vulnerability as learners and their sense of responsibility as emerging professionals.

Subtheme 3.1. Torn between anxiety and anticipation

The participants told that one of their colleagues confessed fear and stress, anger as they heard rumors that clinical practice would be changed hospitals to CTCs due to COVID-19. However, some participants expected to be placed to national crisis response sites where they would participate as students rather than as nurses: “I think I was worried because it was my first experience and there was no one who had experienced it before. But, if you had to go anyway, wouldn’t it be better just to go and experience it?” (P#2); “I had mixed feelings. I was worried, but at the same time, I was excited to try something new at the internship” (P#9).

Subtheme 3.2. Burdened by inexperience and external judgment

The participants expressed that they were not prepared because they were students and that they did not know much, so they felt that they would become a burden at the practice site. Concerns from parents and the media about the dispatch of students without a nursing license to an emergency site for which the treatment was not known led to burdens: “I was a little worried that I might cause problems there because I was not yet a medical professional” (P#3); “I was worried that I would just be a burden to the nurses” (P#5).

Subtheme 3.3. Embracing the call with a sense of purpose

Although the participants were anxious right after the decision to dispatch students to the scene was made, they expressed that they accepted the order with pride in the disaster nursing education, the confidence gained from the preparatory education provided, and the sense of duty when the country needs them: “As a student of nursing school, it is natural to go to the disaster site. The professors provided me with two days of training on what to do before entering the field. I think it was incredibly helpful” (P#6); “I thought it was a special experience because I was a third-year student at the ‘REDACTED’ and was able to participate in it” (P#10).

Theme 4. Validation and pride in becoming visible during a national crisis

Recognition from nurses, the public, and media transformed students’ self-perception- validating their contribution and reinforcing professional identity.

Subtheme 4.1. Recognition from nurses as peers in real practice

The participants felt proud to see a nurse who respected them as a nurse, and not just as a student in clinical practice: “We were in a position of contributing something that nursing workers could check directly and easily, and the fact that our time was very valuable to them made our work more meaningful than just practice” (P#1); “Because we were there from the beginning (of the center), we knew a lot, so we were quickly recognized as being of great help to the nurses” (P#9).

Subtheme 4.2. Public recognition and collective honor

The participants appreciated the social recognition they gained during the clinical practice at the CTCs. The participants felt proud of positive reports in the media about their contribution as students. In addition, although the dispatch was conducted as a clinic practice, they received special duty payment and that made the students feel honorable: “I got hazard pay like a nurse. So, I felt a little prouder of myself for doing this job” (P#8); “After completing the CTCs practice, positive evaluations that we’re more adaptable and communicated better at work compared to other class batches continued” (P#10).

Subtheme 4.3. Gratitude for a chance to fulfil a calling

The participants were grateful that they could contribute to resolving the national crisis. A participant expressed gratitude for the fact that this practice came as an opportunity to participate in solving social problems: “The reason I first decided that I wanted to become a nursing professional was that I wanted to help others. So, the fact that I had the opportunity to do something that can help others came to me as a big opportunity that I am grateful for” (P#7); “I feel proud that I did something more meaningful as a student in a difficult situation at that time” (P#8).

Overall, the four themes depict a trajectory of transformation—from fear and uncertainty to adaptation, professional awareness, and eventual pride. This experiential arc represents the essence of the phenomenon: the process of “becoming” through crisis participation.

## 4. Discussion

This study aimed to illuminate the lived experiences of nursing students who practiced in CTCs during the COVID-19 pandemic, applying Colaizzi’s descriptive phenomenology [19] to reveal the essential meaning structure of those experiences. Through this approach, four interconnected themes emerged, reflecting a dynamic process of emotional endurance, ethical conflict, and professional transformation.

Participants described the CTC practice as a turning point in their professional formation. Consistent with studies that associate crisis participation with identity growth [14,15], this study shows that immersion in a high-risk, socially constrained environment accelerated self-awareness and self-efficacy. However, unlike typical hospital practice, the CTC context forced students to navigate patient care without physical contact, transforming their understanding of empathy and responsibility.

From a phenomenological perspective, this experience aligns with the notion of transformative learning, where encountering a disorienting situation leads to reflection and perspective change. Students reported a newfound confidence-a realization that nursing competence extends beyond technical tasks to emotional and ethical endurance. This finding expands previous literature by situating growth not merely as adaptation but as a profound redefinition of self within the professional role.

Fear of infection, physical confinement, and psychological strain were recurring experiences, echoing prior studies on nurses’ emotional distress during COVID-19 [6,7,8,9,10,11,12,13,14,23]. Yet, this study extends those findings by illustrating how students actively constructed coping mechanisms despite limited autonomy. Through collective living, they built micro-communities of mutual support-moments of laughter, small rituals, and shared endurance—which functioned as protective factors. Moreover, the theme “double restrictions” highlights a novel dimension of role conflict. Students inhabited dual identities: as learners under academic supervision and as quasi-professionals responsible for patient safety. This tension between obedience and autonomy, dependence and responsibility, generated psychological strain but also catalyzed professional maturation. Goode (1960) said the concept of role conflict helps explain the internal negotiation between these roles, offering a lens for understanding stress and identity consolidation in transitional practitioners [24].

Participants’ ambivalence-fear mixed with duty-reveals the ethical and existential dimension of early professional socialization. Similar to findings among nursing students in disaster settings [5,23], the participants experienced moral tension between self-protection and altruism. However, their decision to embrace the assignment demonstrates vocational identity affirmation under crisis. This aligns with the concept of moral resilience, which refers to the capacity to preserve integrity amid ethical complexity [25]. Participants’ narratives suggest that moral resilience was cultivated through structured education, peer solidarity, and the symbolic value of service. In phenomenological terms, this reflects the intentional act of meaning-making—choosing to frame hardship as purposeful contribution. The results also underscore the importance of preparatory ethics education. Experiencing ethical ambiguity firsthand may have strengthened students’ ethical reasoning skills, supporting prior assertions that moral reflection in disaster contexts enhances professional maturity.

The final theme represents the culmination of professional identity formation. Recognition from nurses and the public provided students with external validation, reinforcing their belonging within the nursing community. This differs from conventional clinical placements, where students often remain peripheral observers [26,27]. The phenomenon of becoming visible—being acknowledged as contributors rather than learners—catalyzed a sense of legitimacy and pride. Such validation corresponds to social recognition theory [28] where being recognized by others affirms one’s moral and social worth. Furthermore, this study adds a novel contribution by linking social recognition to identity formation within a crisis. The pride experienced by students was not mere satisfaction; it symbolized the internalization of professional values and the realization of nursing as a social calling. This insight extends the literature by showing how collective honor and visibility in disaster contexts can accelerate the transition from student to professional self-concept.

### Limitations

Limitations of this study include that the data were collected within the initial month after CTCs were founded. Therefore, this study presents insights into the experiences of the early situations in which CTCs began to operate. Among the five CTCs where the clinical practice was performed, the students from only four CTCs were included, so the specificity of all the CTCs were not reflected. And this study used purposive sampling and analyzed the experiences of nursing students at CTCs, so the results cannot be generalized.

## 5. Conclusions

This study explored the lived experiences of nursing students who participated in clinical practice at CTCs during the COVID-19 pandemic. Using Colaizzi’s descriptive phenomenological method, it revealed a trajectory of transformation—from fear and uncertainty to adaptation, ethical reflection, and professional pride.

Students’ immersion in a disaster-care environment provided them with experiential understanding of nursing as both a technical and moral profession. Their narratives showed how vulnerability, responsibility, and recognition interwove to form a new professional self. These findings underscore several implications: (1) Curricular integration: Disaster-preparedness education in nursing should move beyond simulation to include structured, ethically supported field experiences. (2) Ethical and psychological support: Nursing programs deploying students to crisis settings should incorporate continuous supervision, peer support systems, and reflective debriefing to promote moral resilience. (3) Policy development: Policymakers should establish clear guidelines for student involvement in disaster response to safeguard welfare while enabling learning.

## Figures and Tables

**Table 1:** Themes and sub-themes related to the participant’s clinical practice experiences at the CTCs.

	Themes	Sub-Themes
1	Transformative growth through immersive clinical practice in quarantine	Navigating nursing in a non-contact environment
		Stepping into the heart of infectious disease response
		Bearing the emotional weight of quarantine care
		Finding oneself and growing to another level
		Discovering professional identity through crisis
2	Enduring and adapting to uncertainty and emotional turmoil	Overwhelmed by the threat of infection
		Living under double restrictions as students and medical workers
		Anxiety in the face of uncertainty
		Finding moments of relief and connection
		Finding myself to get used to it
3	Reconciling vulnerability and responsibility as future professionals	Torn between anxiety and anticipation
		Burdened by inexperience and external judgment
		Embracing the call with a sense of purpose
4	Validation and pride in becoming visible during national crisis	Recognition from nurses as peers in real practice
		Public recognition and collective honor
		Gratitude for a chance to fulfil a calling

**Table 2:** Participants demographics.

Participant Number	Gender (Age)	Name of CTCs *	Weeks of Practice in CTCs *
P1	Woman (21)	A	3
P2	Woman (21)	A	4
P3	Woman (22)	D	4
P4	Woman (22)	B	4
P5	Woman (22)	B	4
P6	Woman (21)	A	4
P7	Woman (22)	D	4
P8	Woman (22)	B	4
P9	Man (21)	A	4
P10	Woman (22)	C	4

Note. All participants were third-year students who completed 500 h of clinical training and a two-day preparatory education before CTC deployment. * CTCs; Community Treatment Centers.

## Data Availability

The data generated and analyzed during this study are not readily available because ethical approval for use relates only to the research team. Requests to access the datasets should be directed to the corresponding author upon reasonable request, subject to ethical approval.

## Abbreviations

The following abbreviations are used in this manuscript:

CTCs	Community Treatment Centers
COVID-19	The Coronavirus Disease-2019
ICN	International Council of Nurses
